# Multi-centered reassessment of CRS-R in disorders of consciousness: a dimensionality reduction study from cognition and motor function

**DOI:** 10.3389/fneur.2026.1728229

**Published:** 2026-03-18

**Authors:** Qiheng He, Yuhan Shang, Yijun Dong, Tianqing Cao, Xiaoke Chai, Yuanli Zhao, Yi Yang, Ming Song

**Affiliations:** 1 Department of Neurosurgery, Beijing Tiantan Hospital, Capital Medical University, Beijing, China; 2 Eight-Year Program of Clinical Medicine, Peking Union Medical College Hospital, Chinese Academy of Medical Sciences and Peking Union Medical College, Beijing, China; 3 China National Center for Neurological Disorders, Beijing, China; 4 Department of Neurosurgery, Peking Union Medical College Hospital, Chinese Academy of Medical Sciences and Peking Union Medical College, Beijing, China; 5 China National Clinical Research Center for Neurological Diseases, Beijing, China; 6 Beijing Institute of Brain Disorders, Beijing, China; 7 National Laboratory of Pattern Recognition, Institute of Automation, Chinese Academy of Sciences, Beijing, China

**Keywords:** arousal, cognition, disorders of consciousness, motor function, scale

## Abstract

**Objective:**

This study aimed to enhance the Coma Recovery Scale-Revised (CRS-R) for disorders of consciousness (DoC) by developing a two-dimensional model differentiating cognition and motor function.

**Methods:**

We analyzed 124 DoC patients retrospectively and validated findings using five multicenter datasets (*n* = 420). CRS-R subscores were decomposed into Consciousness_x (awareness) and Consciousness_y (arousal/motor function) using Projective Non-negative Matrix Factorization. Logistic regression established diagnostic thresholds, evaluated by accuracy, precision, recall, and F1-score.

**Results:**

The model achieved high accuracy (0.94), precision (0.92), and recall (0.99). Patients with minimally conscious state (MCS) or emerged MCS showed significantly higher scores than vegetative state (VS) patients (*p* < 0.05). The four-quadrant framework revealed distinct clinical profiles: Quadrant I (high awareness/arousal) identified patients for cognitive rehabilitation; Quadrant II (low awareness/high arousal) suggested arousal-enhancing therapies; Quadrant III (low awareness/arousal) indicated VS requiring basic support; Quadrant IV (high awareness/low arousal) highlighted needs for sensorimotor integration.

**Conclusions:**

The two-dimensionally reduced representation of CRS-R scores maintains diagnostic accuracy while improving DoC classification. The four-quadrant model enables personalized interventions.

**Trial registration:**

Our study has been verified by the Chinese Clinical Trial Registry with the registration number: ChiCTR2400085855, and the registration date is June 19, 2024.

## Introduction

Disorders of consciousness (DoC) refer to a disturbance state of consciousness occurring in patients resulting from severe brain injuries, neurological disorders, or other medical conditions. These conditions are characterized by varied levels of wakefulness and awareness, including coma, vegetative state (VS), and minimally conscious state (MCS) (1–4). This condition poses profound threats to the patient's arousal, awareness, and motor functions and poses profound challenges to medical management and patient rehabilitation (2, 5, 6). In clinical practice, the most exhaustive and evidence-based behavioral assessment tool for identifying indicators of consciousness in patients undergoing coma recovery is the Coma Recovery Scale-Revised (CRS-R) (7, 8). CRS-R is a comprehensive assessment tool with six subscales, differentiating between coma [defined by the absence of arousal and awareness (9)], the vegetative state [VS, defined by the presence of arousal without awareness (10, 11)], minimally conscious state [MCS, defined by minimal, reproducible, but inconsistent awareness (12)], and emergence from MCS (eMCS) (13).

However, CRS-R scores have limitations, such as the inability to reflect underlying causes and the lack of detailed prognostic information, and the total score alone cannot effectively differentiate between the relative impairment across different functional dimensions. Patients with similar overall scores may present different clinical manifestations, and their treatments may require different approaches, posing challenges in treatment decisions (14, 15). However, the clinical diagnosis using CRS-R primarily relies on a categorical “present/absent” judgment of specific behavioral items. While clear, this approach has limitations: First, it cannot quantify the relative impairment across core functional dimensions (e.g., awareness vs. arousal-motor function), which may have distinct neural substrates. Second, for patients with total scores in the “diagnostic gray zone” (e.g., 4–9), heterogeneous subscore profiles are grouped into the same category, potentially obscuring different pathophysiologies and rehabilitation potentials. Therefore, a tool to continuously parse the CRS-R profile is needed for finer phenotyping. Neuroscience and clinical assessment of DoC often consider three key interconnected functional domains arousal, awareness, and motor functions. Arousal takes precedence in neurological assessments, involving the coordinated functioning of the brainstem and cerebral cortex. The ability of patients to spontaneously maintain wakefulness directly reflects the fundamental physiological status of the nervous system (16). Preliminary assessments of arousal involve observing whether patients can spontaneously open their eyes, sustain wakefulness, and appropriately respond to external stimuli (17). Awareness pertains to the perception and understanding of external stimuli and one's own state. This dimension is directly correlated with a patient's comprehension of language, visual, and auditory stimuli, forming the foundation for cognitive interaction with the external environment. Impairments in awareness may lead to a sense of confusion or disorientation regarding the surrounding world, significantly impacting the overall quality of life and rehabilitation of patients (18). The motor function dimension focuses on whether patients can spontaneously engage in motor activities, encompassing muscle coordination and motor control. These three dimensions exhibit intricate interrelationships.

Despite the assistance provided by neuroimaging and neurophysiological tools, precise dimensional stratification starting from clinical etiology and behavioral manifestations remains challenging. Previous attempts to simplify or elaborate CRS-R scoring, such as using the total score for prognosis (19), have shown utility but do not explicitly disentangle these core dimensions. Therefore, we proposed an innovative approach of reducing the CRS-R scores to two core, data-driven dimensions. This reduction aims to decompose the complex behavioral profile into more fundamental components of awareness and combined arousal/motor function, potentially offering a clearer assessment of patient deficits and a foundation for targeted interventions.

## Materials and methods

### Study subjects data collection

The retrospective study analyzed patients who were admitted at the Department of Disorders of Consciousness, The Seventh Medical Center of the General Hospital of the People's Liberation Army of China between January 2018 and December 2021. The inclusion criteria were: (1) Meet the diagnosis of chronic DoC according to Multi-Society Task Force on the Persistent Vegetative State (33). (2) The patients' neurological function showed no significant progress for 3 months prior to enrollment. (3) Informed consent has been obtained from the patient. The exclusion criteria were: (1) The patient does not consent to the collection of information for this study. The study was approved by the ethics committee of Beijing Tiantan Hospital, Capital Medical University (KY2023-161-03).

Ethical Compliance for Multi-center Data: The retrospective data from The Seventh Medical Center of the General Hospital of the People's Liberation Army of China were collected in accordance with its institutional guidelines. The protocol for this retrospective analysis, including the use of these data and the publicly available datasets from published studies, was reviewed and approved by the Institutional Review Board of Beijing Tiantan Hospital, Capital Medical University (KY2023-161-03), which served as the ethical oversight body for this specific research project. All publicly cited datasets were obtained from previously published studies where informed consent had been acquired by the original authors.

Six data sets were included in this study, with one test dataset collected at our center and five validation datasets from previous articles between 2014 and 2020 (20–24). Baseline variables, including age, sex, pathogeny, and behavioral evaluation [Chinese translation of the Coma Recovery Scale-Revised (CRS-R)] were extracted (25, 26). The CRS-R includes six subscales that address auditory, visual, motor, oromotor, communication, and arousal functions, which are summed to yield a total score ranging from 0 to 23. In our center's assessment, we considered the highest value obtained from three measurements within 1 week as representing the patient's level of consciousness.

### Two-dimensional reduction of the CRS-R structure

We used non-negative matrix factorization (NMF) to analyze the data structure of CRS-R. In this study, each patient was considered as a sample. Since the CRS-R score of each patient consisted of six sub-scores, we modeled each sample as a six-dimensional vector. The NMF can decompose the original data into interpretable components, which can enhance the clarity and understanding of the underlying structure of the data. Importantly, NMF ensures that both the input matrix and the factors it decomposes into are non-negative (27). This leads to more interpretable results, which can be crucial for the analysis of data structure of the clinical scale.

Specifically, this study employed the Projective Non-negative Matrix Factorization (PNMF) method (28). PNMF projects high-dimensional non-negative data X onto a lower-dimensional subspace spanned by a non-negative basis W and considers WT X as their coefficients, i.e., X≈WWT X (29). The advantages of this method include: fewer parameters, low computational complexity, good generalization, sparse components, and a certain degree of orthogonality. In addition, the PNMF demonstrated the good ability for clustering, especially for high-dimensional data (30). The selection of Projective Non-negative Matrix Factorization (PNMF) as the core dimensionality reduction method in this study was primarily based on its unique advantages for handling clinical scale data. Firstly, the non-negativity constraint inherent to PNMF ensures that the component weights obtained from the factorization are all non-negative values. This aligns naturally with the non-negative nature of the CRS-R sub-scores (range 0–3), conferring intuitive clinical interpretability to the ultimately extracted dimensions (Consciousness_x and Consciousness_y)—each dimension can be understood as a non-negative linear combination of several sub-scores, readily interpretable as “contributions.” In contrast, methods like Principal Component Analysis (PCA) may produce loadings with negative values, which are difficult to directly interpret in biological or clinical terms within the context of behavioral scales. Secondly, PNMF has been demonstrated to excel in clustering high-dimensional data; its objective function implicitly promotes a clustered structure of samples in the low-dimensional space, which aligns precisely with this study's core objective of differentiating distinct states of consciousness (VS, MCS). To preliminarily validate the suitability of PNMF in this context, we performed a comparative analysis against PCA (results not detailed in the main text but available upon request). In brief, on the same validation set, the classifier constructed using the two dimensions extracted by PNMF demonstrated superior classification accuracy and clustering separability (as measured by the between-class to within-class variance ratio) compared to the model built using the first two principal components from PCA. Therefore, PNMF represents an appropriate choice for this study, balancing result interpretability with superior discriminatory efficacy.

Since two-dimensional representations allows for easier visualization and interpretation of relationships among variables or samples, especially in the clinical practices, this study set the number of PNMF components to two. This means that this study used two components to approximate the DoC patients' CRS-R scores data that previously required six dimensions for representation.

### Logistic regression model for diagnosis

To establish diagnostic boundaries between VS and MCS based on the two extracted dimensions, we trained two logistic regression models (one for each dimension) using the glm function in R (version 4.1.1) (R Foundation for Statistical Computing, Vienna, Austria). The decision boundaries (vertical and horizontal thresholds in the two-dimensional space) were derived from the model coefficients. The performance of the combined two-dimensional classifier was evaluated on the validation set using accuracy, precision, recall, and F1-score. Additionally, the diagnostic performance using each single dimension individually was also calculated for comparison.

### Validations of the usability of the two-dimensional reduction for the diagnosis of DoC

To validate the usability for the diagnosis of DoC, this study applied the two-dimensional projective matrix to the CRS-R scores of the DoC patients which were reported in the previous literature. We examined numerous literature sources and found that five articles provided all of the six CRS-R subscales for their involved DoC patients (20–24). The five studies from different medical centers in Europe or Asia included a total of 362 DoC patients. Notably, Schorr et al. (23) provided both the baseline and 12 months follow-up CRS-R scores of the 58 patients with DoC.

### Statistical analysis

R (version 4.1.1) software was used for data statistics and analysis. Normally distributed or non-normally distributed continuous variables are presented as mean ± standard deviation or median (IQR), respectively. The homogeneity of variance was tested. According to the variance, chi-square test or two-way repeated measures analysis was used. *P* < 0.05 was considered to be significantly different. The function geom_jitter was used to avoid the overlap between different points.

## Results

### Study population

This multicenter study enrolled 486 patients with disorders of consciousness (DoC), comprising 124 patients in the training set and 420 in the validation set. Consequently, the model was constructed without exposure to the phenotypic patterns of eMCS patients, which may influence its performance in discriminating the highest levels of consciousness within the DoC spectrum. Despite this, the model was evaluated on independent validation sets that included eMCS cases. As presented in Table 1, the overall population had a mean age of 45.8 ± 16.4 years (range across cohorts: 37.9–51.6 years) with male predominance (62.3%, range: 52.4%−74.4%).

**Table 1:** Baseline characteristics in patients with DoC^a^ across centers.

**Characteristic**	**Training (*N* = 124)**	**Validation (*****N*** = **420)**					
		**Sitt et al. (24) (*N* = 113)**	**Demertizi et al. (20) (*N* = 48)**	**Demertizi et al. (21) (*N* = 65)**	**Schorr et al. (23) (on admission, *N* = 58)**	**Schorr et al. (23) (follow up, *N* = 58)**	**Pan et al.22 (*N* = 78)**
Age, y, mean (SD^b^)	43.1 ± 15.1	48.2 ± 17.5	47.6 ± 17.2	45.0 ± 17.1	51.6 ± 16.1	51.6 ± 16.1	37.9 ± 15.3
Sex, male, *n* (%)	65 (52.4)	79 (69.9)	30 (62.5)	44 (67.7)	38 (65.5)	38 (65.5)	51 (65.4)
**Pathogeny**, ***n*** **(%)**							
Anoxia	42 (33.9)	32 (28.3)	11 (22.9)	18 (27.7)	24 (41.4)	24 (41.4)	0 (0)
Stroke	43 (34.7)	38 (33.6)	14 (29.2)	20 (30.8)	15 (25.9)	15 (25.9)	20 (25.6)
Trauma	35 (28.2)	24 (21.2)	21 (43.8)	24 (36.9)	16 (27.6)	16 (27.6)	58 (74.4)
Other	4 (3.2)	19 (16.8)	2 (4.2)	3 (4.6)	3 (5.2)	3 (5.2)	0 (0)
**Diagnosis**, ***n*** **(%)**							
VS/UWS^c^	98 (79.0)	75 (66.4)	24 (50.0)	24 (36.9)	57 (98.3)	37 (63.8)	70 (89.7)
MCS^d^	26 (21.0)	67 (59.3)	24 (50.0)	40 (61.5)	1 (1.7)	8 (13.8)	62 (79.5)
eMCS^e^	0 (0)	25 (22.1)	0 (0)	1 (1.5)	0 (0)	4 (6.9)	15 (19.2)
Preoperative CRS-R^f^, point, mean (SD)	6.9 ± 2.7	9.2 ± 5.6	9.8 ± 3.0	8.2 ± 3.8	5.5 ± 2.9	6.9 ± 4.8	8.9 ± 3.0

The detailed characteristics of comprising 124 patients in the training set and 420 in the validation set.

^a^DoC, Disorders of consciousness.

^b^SD, standard deviation.

^c^VS/UWS, vegetative state/unresponsive wakeness state.

^d^MCS, minimally conscious state.

^e^eMCS, emerged minimally conscious state.

f, CRS-R, coma recovery scale-revised.

Slight difference was observed across cohorts: anoxic brain injury (0%−41.4%), stroke (25.6%−34.7%), and traumatic brain injury (21.2%−74.4%). Diagnostic distribution demonstrated substantial variability, with vegetative state/unresponsive wakefulness syndrome (VS/UWS) accounting for 36.9%−98.3% at baseline assessment, while minimally conscious state (MCS) prevalence ranged from 1.7% to 61.5%. The validation cohorts showed higher proportions of emerged MCS (eMCS) cases (up to 22.1%), whereas no eMCS cases were identified in the training set.

### CRS-R score using non-negative matrix factorization in patients with DoC

Among 124 patients with DoC enrolled at our center, we applied PNMF to factorize the six subscales of CRS-R scores. According to the methods described above, the top two components in the non-negative matrix are as follows:


$$\begin{array}{r} Consciousness\text{\_}x\;=0.276\times Auditory+0.895\times Visual+0.000\\ \times Motor+0.000\times Oral+0.041\times Communication\\ +\;0.000\times Arousal\\ Consciousness_{y}=0.240\times Auditory+0.000\times Visual+0.705\\ \times Motor+0.294\times Oral+0.000\times Communication\\ +\;0.568\times Arousal \end{array}$$

**Figure 1:**
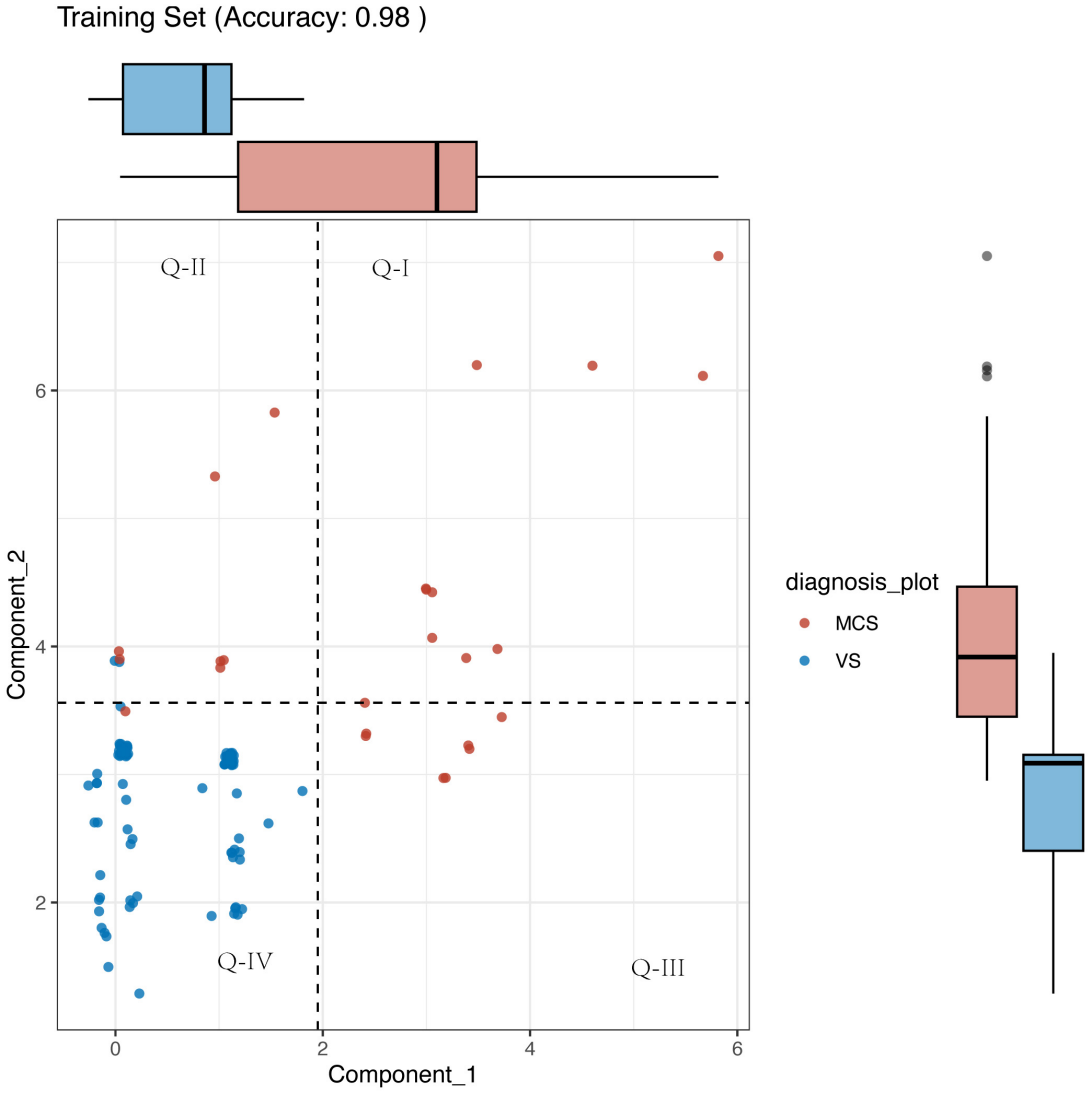
Two-dimensional representation of patients in the training set. The plot illustrates the projection of Coma Recovery Scale-Revised (CRS-R) subscores onto the two principal components derived from Projective Non-negative Matrix Factorization (PNMF): Consciousness_x (awareness, x-axis) and Consciousness_y (arousal/motor function, y-axis). Patients are color- and shape-coded by diagnosis: Minimally Conscious State (MCS) is represented by blue triangles (Δ), and Vegetative State (VS) by gray circles (°). The dashed horizontal and solid vertical lines represent the decision boundaries derived from logistic regression, dividing the plane into four quadrants (Q-I to Q-IV). The clear separation of clusters visualizes the model's high discriminatory power between VS and MCS.

Consciousness_x is weighted predominantly by the visual and auditory subscales, which are core indicators of awareness. Consciousness_y is weighted predominantly by the motor, arousal, and oromotor subscales, representing a composite dimension of arousal and motor function. The high weighting of the visual subscale in Consciousness_x indicates it was the most discriminative single indicator for the latent “awareness” factor in our dataset, likely due to its objective assessment. We thus clinically interpret this dimension as “Awareness.” The Consciousness_y dimension, dominated by motor and arousal subscores, is interpreted as a composite “Arousal/Motor function” dimension. This factorization aligns with the dual-system framework of consciousness (cortical integration) and arousal/motor preparedness (subcortical circuits). Based on the formula derived from the above component decomposition, we computed the scores of DoC patients under the two new components, as shown in Figure 1.

The specific weighting pattern of the components, particularly the prominent role of the visual subscale, is further interpreted in the Discussion section.

### Classification statistics of the awareness and arousal/motor function dimensions in the training dataset

The decision tree boundaries between VS and MCS are illustrated in the Figure 1. The model exhibits high accuracy (0.98), precision (0.99) and recall (0.98), indicating strong performance in distinguishing between the two states. Figure 1 presents a clear visualization of the decision boundaries, with MCS patients predominantly occupying the upper and right regions of the plot, while VS patients are mainly located in the lower and left regions. This distinction is further supported by the significantly higher median and broader distribution range of both components for MCS patients.

### Classification statistics of the awareness and arousal/motor function dimensions in the validation dataset

**Figure 2:**
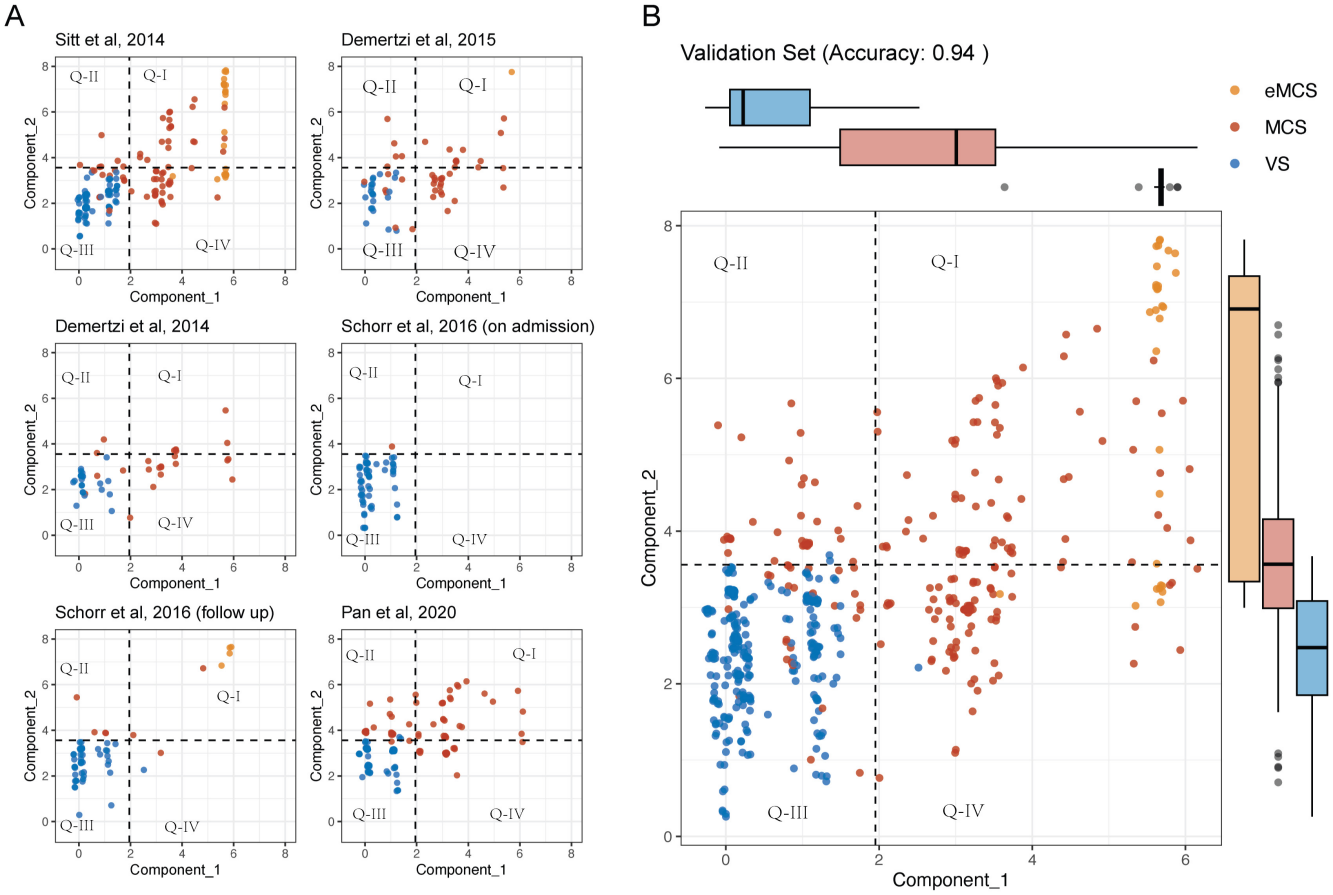
**(A)** Two-dimensional representation of Consciousnessx and Consciousnessy in the training set (*n* = 124). **(B)** Scatter plot of the validation set (*n* = 420) with patients color-coded by diagnosis: eMCS (orange), MCS (red), VS (blue). Box plots on the top and right margins show the distribution of Consciousnessx and Consciousnessy across groups. The model achieved an accuracy of 0.94.

Using the aforementioned formula, we aimed to project each patient's CRS-R subscales into two components. The results demonstrated that this representation could effectively cluster patients with different diagnostic categories of DoC across multiple independent datasets, as shown in Figure 2. The consolidated validation data from these studies demonstrated high accuracy (0.94), precision (0.92), and recall (0.99), further confirming the robustness of our approach. Statistical analysis revealed that both the Consciousness_x and Consciousness_y dimensions were significantly higher in eMCS compared to MCS (*p* < 0.05), and higher in MCS compared to VS (*p* < 0.05). These findings underscore the importance of both dimensions in accurately classifying VS and MCS.

### Interpretation of wrong classifications

Despite the high overall accuracy, some patients were misclassified into incorrect categories. We examined these cases in detail. Table 2 lists all misclassified patients and summarizes their characteristics, highlighting the need for reassessment.

**Table 2:** Subscores of DoC^a^ patients being incorrectly classified.

**Patients no**.	**Total**	**Auditory**	**Visual**	**Motor**	**Oralmotor**	**Communication**	**Arousal**	**Diagnosis**
1	6	3	0	2	1	0	0	MCS^b^
2	8	3	0	2	1	0	2	MCS
3	4	0	2	0	1	0	1	MCS
4	7	3	0	1	2	0	1	MCS
5	9	3	0	2	2	1	1	MCS
6	5	0	0	3	1	0	1	MCS
7	5	4	0	0	0	1	0	MCS
8	5	1	0	1	1	1	1	MCS
9	8	3	1	1	1	0	2	MCS
10	7	3	0	1	2	0	1	MCS
11	7	1	1	3	0	0	2	MCS
12	9	2	1	2	2	0	2	VS^c^
13	9	2	1	2	2	0	2	VS
14	8	3	0	2	1	0	2	MCS
15	8	3	0	2	1	0	2	MCS
16	7	2	0	3	1	0	1	MCS
17	9	3	0	2	2	1	1	MCS
18	9	3	1	2	1	0	2	MCS
19	6	3	0	1	1	0	1	MCS
20	8	3	1	2	1	0	1	MCS
21	8	2	1	3	1	0	1	MCS
22	9	3	1	2	1	1	1	MCS
23	9	3	1	1	2	0	2	MCS
24	6	3	0	1	1	0	1	MCS
25	7	1	1	3	1	0	1	MCS
26	6	3	0	1	1	0	1	MCS
27	6	1	1	0	1	1	2	MCS
28	9	2	0	3	3	1	0	MCS
29	7	2	2	1	0	0	2	VS

The outcomes and corresponding diagnoses of 29 patients were evaluated across six dimensions.

^a^DoC, Disorders of consciousness.

^b^MCS, minimally conscious state.

^c^VS/UWS, vegetative state/unresponsive wakeness state.

Few cases present impossible or improbable combinations, suggesting potential errors during evaluation and interpretation. For example, an unarousable patient (arousal = 0) with consistent command following (auditory = 4) was misclassified, possibly due to conditions such as bilateral ptosis, facial edema, or eyelid apraxia (1).

## Discussion

Our study introduces a novel approach to assessing patients with Disorders of Consciousness (DoC) by reducing the Coma Recovery Scale-Revised (CRS-R) scores to two primary dimensions: arousal/motor function and awareness (31). This two-dimensional reduction simplifies the traditionally complex six-dimensional assessment into a more clinically intuitive two-dimensional model. This model serves as a complementary tool that maintains the diagnostic accuracy of the original CRS-R while providing a quantitative profile of the relative impairment in awareness and arousal/motor function, which may enhance phenotypic differentiation and inform personalized treatment strategies. These extracted dimensions are grounded in the established neuroscience of consciousness. Arousal and awareness are fundamental components in the assessment of consciousness. Arousal, primarily maintained by brainstem and subcortical structures, reflects a patient's capacity to stay awake and respond to stimuli. Awareness, which depends on cortical and higher subcortical functions, involves the ability to perceive and interact with the environment. By decomposing CRS-R scores into these two dimensions, we can more precisely evaluate the functional state of DoC patients. It is noteworthy that within the Consciousness_x component, the visual subscale contributed a predominant weight (0.895), while motor, oromotor, and arousal subscales had zero weights. This outcome indicates that, within our dataset, behavioral responses in the visual domain served as the most sensitive and discriminative single indicator for differentiating levels of “awareness” captured by this data-driven model. This may be attributed to several factors: (1) visual behaviors such as visual pursuit and fixation are assessed with relatively high objectivity and reproducibility in the CRS-R; (2) the recovery of visual function may exhibit strong collinearity with the recovery of other cognitive faculties (e.g., auditory comprehension) along the spectrum of consciousness recovery, thus being statistically captured as the primary signal. However, this model-derived weighting does not imply that awareness is solely dependent on visual function in clinical practice. Auditory command following, communication, and other behaviors remain cornerstone measures for assessing consciousness. This result highlights the data-driven nature of the model and underscores that its interpretation should be integrated with comprehensive clinical assessment.

The decomposition of the CRS-R into these two continuous dimensions, despite the prominent role of visual subscores in the awareness component, provides a structured and simplified framework that clarifies the relative contributions of different behavioral modalities to the overall clinical picture.

The core advance of this study is the provision of a continuous, two-dimensional coordinate system that complements categorical CRS-R diagnosis. This model outperforms diagnostic approaches relying solely on the total CRS-R score or single dimensions (as supported by our validation results), demonstrating that disentangling awareness from arousal/motor function captures non-redundant, clinically meaningful variance.

The observed heterogeneity in etiological distribution across cohorts may be attributed to several factors. First, differences in patient recruitment sources likely contributed to the variability—tertiary referral centers may receive more severe trauma cases, while rehabilitation facilities might enroll more anoxic injury patients. Second, regional epidemiological patterns (e.g., varying stroke prevalence) and institutional admission criteria could further explain the divergent proportions of traumatic (21.2%−74.4%) vs. anoxic (0%−41.4%) injuries.

Regarding diagnostic distribution differences, the wide ranges in VS/UWS (36.9%−98.3%) and MCS (1.7%-61.5%) frequencies may reflect: (1) Methodological limitations : Smaller sample sizes (particularly in cohorts with *N* < 100) increase susceptibility to sampling bias. (2) Temporal factors : Later studies [e.g., (22)] showed higher eMCS rates (19.2%), possibly indicating improved diagnostic sensitivity over time. (3) Assessment protocols: Variability in CRS-R administration timing (acute vs. chronic phase) may influence syndrome classification.

By employing PNMF, our approach seeks to decompose the multidimensional CRS-R data into two distinct components—arousal/motor function and awareness (32). This dimensionality reduction approach aims to reduce the dimensionality of the data while preserving the essential characteristics that differentiate VS, MCS, and eMCS. Our findings indicate that the two-dimensional model effectively separates patients into four quadrants, each representing distinct clinical profiles. The quadrant boundaries are defined by the diagnostic thresholds for VS/MCS on each dimension, thus creating a phenotypic map rather than an arbitrary division. Patients in the third quadrant exhibit poor arousal and awareness, aligning with the characteristics of the VS. In contrast, those in the first quadrant demonstrate higher levels of both dimensions, approaching a conscious state, indicative of MCS or better. The second quadrant includes patients with good awareness but poor arousal, while the fourth quadrant contains patients with good arousal but limited awareness, both requiring specialized clinical attention. Through this method, we propose a more targeted assessment model that could potentially enhance both diagnostic accuracy and therapeutic relevance by clearly delineating the contributions of arousal and awareness in patient scores. Building upon the two-dimensional diagnostic space, we further propose a theoretically-derived, four-quadrant interventional framework as a hypothesis for personalized care. This framework maps each quadrant to a potential therapeutic focus, providing a structured starting point for clinical reasoning. However, it is crucial to emphasize that the efficacy and superiority of interventions based on this specific framework have not been validated within the present study, which was primarily diagnostic and retrospective in nature. The framework itself represents a key hypothesis generated from our findings, and its validation constitutes an essential next step for future research.

Building upon the two-dimensional space, we derive a hypothetical four-quadrant intervention framework to generate structured clinical hypotheses. For example, Quadrant I (high awareness/arousal) might prioritize cognitive rehabilitation, while Quadrant IV (high arousal/low awareness) could target sensory integration. Critically, this therapeutic mapping is a theoretical derivation. Its clinical efficacy and predictive value require prospective validation in interventional studies.

Furthermore, the proposed four-quadrant intervention framework, while conceptually useful, remains a hypothesis-generating model. Its clinical utility in predicting differential treatment responses needs rigorous validation through prospective trials.

The analysis of misclassifications provided critical insights. The 29 misclassified patients all possessed original CRS-R total scores clustered within the 4–9 “gray zone” (Table 2). This strongly demonstrates that the primary source of diagnostic error for the conventional method lies in this ambiguous score range, where patients with similar total scores but divergent subscore profiles (e.g., high auditory/low visual vs. low auditory/high visual) are lumped together. Our two-dimensional model directly addresses this limitation by disentangling the underlying contributions of awareness (Consciousness_x) and arousal/motor function (Consciousness_y). By doing so, it provides a finer-grained discrimination within this problematic range, thereby resolving ambiguities inherent to the traditional one-dimensional total score approach. This capability represents a key clinical advantage of the model.

The primary advantage of our approach lies in its simplicity and clarity, making it easier for clinicians to interpret and apply in practice. The high accuracy of the PNMF model in distinguishing between VS and MCS underscores its potential as a reliable diagnostic tool.

However, this study has several limitations that should be considered. First and foremost, the training dataset contained no eMCS cases. While the model demonstrated an ability to differentiate eMCS from MCS and VS in independent validation sets, its construction did not incorporate the full phenotypic spectrum of consciousness recovery. This absence may affect the precision of the model in distinguishing nuanced differences between high-level MCS and eMCS, and future iterations should be trained on datasets encompassing the entire DoC continuum. Second, the study was retrospective rather than prospective in design. Third, the sample size, though validated across multiple centers, remains moderate. In future research, we plan to enroll more patients, particularly those with eMCS, and incorporate advanced imaging techniques and electrophysiological technologies in a prospective design. but also enhance our understanding of the temporal dynamics of the awareness and arousal/motor function dimensions.

## Conclusion

This study demonstrates that representing CRS-R scores along the two continuous dimensions of arousal and awareness maintains diagnostic accuracy while significantly improving clinical differentiation of disorders of consciousness (DoC) states. The established four-quadrant model not only resolves classification ambiguities in borderline cases inherent to conventional CRS-R scoring but more importantly, and proposes a theoretically-grounded, clinically actionable intervention framework that warrants prospective validation.

## Data Availability

The raw data supporting the conclusions of this article will be made available by the authors, without undue reservation.

## References

[1] Chatelle C Bodien YG Carlowicz C Wannez S Charland-Verville V Gosseries O . Detection and interpretation of impossible and improbable coma recovery scale-revised scores. Arch Phys Med Rehabil. (2016) 97:1295–300.e4. doi: 10.1016/j.apmr.2016.02.009 26944708 PMC6095641

[2] Giacino JT Katz DI Schiff ND Whyte J Ashman EJ Ashwal S . Practice guideline update recommendations summary: disorders of consciousness: report of the guideline development, dissemination, and implementation subcommittee of the American academy of neurology; the american congress of rehabilitation medicine; and the national institute on disability, independent living, and rehabilitation research. Neurology. (2018) 91:450–60. doi: 10.1212/WNL.0000000000005926 30089618 PMC6139814

[3] Kondziella D Bender A Diserens K Van Erp W Estraneo A Formisano R . European academy of neurology guideline on the diagnosis of coma and other disorders of consciousness. Eur J Neurol. (2020) 27:741–56. doi: 10.1111/ene.14151 32090418

[4] Zhang X Zhang R Xiong F Zhang Y Li Y. Consciousness disorders and swallowing difficulties. Dysphagia. (2025) 40:1275–81. doi: 10.1007/s00455-025-10834-2 40379843 PMC12662887

[5] Eapen BC Georgekutty J Subbarao B Bavishi S Cifu DX. Disorders of consciousness. Phys Med Rehabil Clin N Am. (2017) 28:245–58. doi: 10.1016/j.pmr.2016.12.003 28390511

[6] Edlow BL Claassen J Schiff ND Greer DM. Recovery from disorders of consciousness: mechanisms, prognosis and emerging therapies. Nat Rev Neurol. (2021) 17:135–56. doi: 10.1038/s41582-020-00428-x 33318675 PMC7734616

[7] Bodien YG Katz DI Schiff ND Giacino JT. Erratum: behavioral assessment of patients with disorders of consciousness. Semin Neurol. (2022) 42:e1–e1. doi: 10.1055/s-0043-1775572 37903645

[8] Giacino JT Kalmar K Whyte J. The JFK coma recovery scale-revised: measurement characteristics and diagnostic utility. Arch Phys Med Rehabil. (2004) 85:2020–9. doi: 10.1016/j.apmr.2004.02.033 15605342

[9] Teasdale G Jennett B. Assessment of coma and impaired consciousness. Lancet. (1974) 304:81–4. doi: 10.1016/S0140-6736(74)91639-0 4136544

[10] Jennett B Plum F. Persistent vegetative state after brain damage. Lancet. (1972) 299:734–7. doi: 10.1016/S0140-6736(72)90242-5 4111204

[11] The The European Task Force on Disorders of Consciousness Laureys S Celesia GG Cohadon F Lavrijsen J León-Carrión J . Unresponsive wakefulness syndrome: a new name for the vegetative state or apallic syndrome. BMC Med. (2010) 8:68. doi: 10.1186/1741-7015-8-68 21040571 PMC2987895

[12] Giacino JT Ashwal S Childs N Cranford R Jennett B Katz DI . The minimally conscious state: definition and diagnostic criteria. Neurology. (2002) 58:349–53. doi: 10.1212/WNL.58.3.349 11839831

[13] Bodart O Laureys S Gosseries O. Coma and disorders of consciousness: scientific advances and practical considerations for clinicians. Semin Neurol. (2013) 33:83–90. doi: 10.1055/s-0033-1348965 23888393

[14] Bai Y. Opportunities and challenges in the diagnosis and treatment of disorders of consciousness. Brain Sci. (2025) 15:487. doi: 10.3390/brainsci15050487 40426658 PMC12109859

[15] Bodien YG Allanson J Cardone P Bonhomme A Carmona J Chatelle C . Cognitive motor dissociation in disorders of consciousness. N Engl J Med. (2024) 391:598–608. doi: 10.1056/NEJMoa2400645 39141852 PMC7617195

[16] Laureys S Faymonville M Luxen A Lamy M Franck G Maquet P. Restoration of thalamocortical connectivity after recovery from persistent vegetative state. Lancet. (2000) 355:1790–1. doi: 10.1016/S0140-6736(00)02271-6 10832834

[17] Plum F Posner JB. The Diagnosis of Stupor and Coma. Oxford University Press (1982). Available online at: https://books.google.com.sg/books?id=Pbl4CH4NlQsC (Accessed March 12, 2026).

[18] Giacino JT Fins JJ Laureys S Schiff ND. Disorders of consciousness after acquired brain injury: the state of the science. Nat Rev Neurol. (2014) 10:99–114. doi: 10.1038/nrneurol.2013.279 24468878

[19] Bodien YG Carlowicz CA Chatelle C Giacino JT. Sensitivity and specificity of the coma recovery scale–revised total score in detection of conscious awareness. Arch Phys Med Rehabil. (2016) 97:490-1. doi: 10.1016/j.apmr.2015.08.422 26342571 PMC5018674

[20] Demertzi A Gómez F Crone JS Vanhaudenhuyse A Tshibanda L Noirhomme Q . Multiple fMRI system-level baseline connectivity is disrupted in patients with consciousness alterations. Cortex. (2014) 52:35–46. doi: 10.1016/j.cortex.2013.11.005 24480455

[21] Demertzi A Antonopoulos G Heine L Voss HU Crone JS De Los Angeles C . Intrinsic functional connectivity differentiates minimally conscious from unresponsive patients. Brain. (2015) 138:2619–31. doi: 10.1093/brain/awv169 26117367

[22] Pan J Xie Q Qin P Chen Y He Y Huang H . Prognosis for patients with cognitive motor dissociation identified by brain-computer interface. Brain. (2020) 143:1177–89. doi: 10.1093/brain/awaa026 32101603 PMC7174053

[23] Schorr B Schlee W Arndt M Bender A. Coherence in resting-state EEG as a predictor for the recovery from unresponsive wakefulness syndrome. J Neurol. (2016) 263:937–53. doi: 10.1007/s00415-016-8084-5 26984609

[24] Sitt JD King J-R El Karoui I Rohaut B Faugeras F Gramfort A . Large scale screening of neural signatures of consciousness in patients in a vegetative or minimally conscious state. Brain. (2014) 137:2258–70. doi: 10.1093/brain/awu141 24919971 PMC4610185

[25] Di H He M Zhang Y Cheng L Wang F Nie Y . Chinese translation of the coma recovery scale—Revised. Brain Injury. (2017) 31:363–5. doi: 10.1080/02699052.2016.1255780 28125307

[26] Zhang Y Wang J Schnakers C He M Luo H Cheng L . Validation of the Chinese version of the coma recovery scale-revised (CRS-R). Brain Injury. (2019) 33:529–33. doi: 10.1080/02699052.2019.1566832 30663434

[27] Lee DD Seung HS. Learning the parts of objects by non-negative matrix factorization. Nature. (1999) 401:788–91. doi: 10.1038/44565 10548103

[28] Yuan Z Oja E. Projective nonnegative matrix factorization for image compression and feature extraction. In:Kalviainen H Parkkinen J Kaarna A, editors. Image Analysis, Vol. 3540. Berlin, Heidelberg: Springer (2005). p. 333–42. doi: 10.1007/11499145_35

[29] Guan N Zhang X Luo Z Tao D Yang X. Discriminant projective non-negative matrix factorization. PLoS ONE. (2013) 8:e83291. doi: 10.1371/journal.pone.0083291 24376680 PMC3869764

[30] Yang Z Oja E. Linear and nonlinear projective nonnegative matrix factorization. IEEE Trans Neural Netw. (2010) 21:734–49. doi: 10.1109/TNN.2010.2041361 20350841

[31] Wang L Liu C Lu E Zhang D Zhang H Xu X . Total intracranial volume as a covariate for predicting prognosis in patients with primary intracerebral hemorrhage. Clin Neurol Neurosurg. (2022) 214:107135. doi: 10.1016/j.clineuro.2022.107135 35121234

[32] Zhou M Carin L. Negative binomial process count and mixture modeling. IEEE Trans Pattern Anal Mach Intell. (2015) 37:307–20. doi: 10.1109/TPAMI.2013.211 26353243

[33] Multi-Society Task Force on PVS. Medical aspects of the persistent vegetative state (First of Two Parts). N Engl J Med. (1994) 330:1499–508. doi: 10.1056/NEJM199405263302107 7818633

